# Bridging therapy before CAR-T for multiple myeloma: a survey from the CMWP and CTIWP of the EBMT

**DOI:** 10.1038/s41409-026-02816-1

**Published:** 2026-03-31

**Authors:** Nico Gagelmann, Maximilian Merz, Laurien G. A. Baaij, Linda Koster, Jorinde D. Hoogenboom, Joanna Drozd-Sokolowska, Kavita Raj, Jürgen Kuball, Patrick J. Hayden, Florent Malard, Laurent Garderet, Annalisa Ruggeri, Donal McLornan

**Affiliations:** 1 University Medical Center Hamburg-Eppendorf, Hamburg, Germany; 2 University Hospital Leipzig, Leipzig, Germany; 3 Memorial Sloan Kettering Center, New York, NYC USA; 4 EBMT Leiden Study Unit, Leiden, the Netherlands; 5 University Clinical Centre, Medical University of Warsaw, Warsaw, Poland; 6 University College London Hospitals NHS Trust, London, UK; 7 University Medical Centre Utrecht, Utrecht, the Netherlands; 8 Department of Haematology, Trinity College Dublin, St. James’s Hospital, Dublin, Ireland; 9 Hôpital Saint-Antoine, AP-HP, Paris, France; 10 Hôpital Pitié Salpêtrière, Hematology department, Paris, France; 11 IRCCS San Raffaele, Milano, Italy

**Keywords:** Disease-free survival, Epidemiology

## To the Editor:

Consequently, most candidates for anti-BCMA CAR-T receive “bridging therapy” to control disease and maintain performance status while awaiting infusion [1]. However, practices remain highly individualized, with choices driven by disease tempo, prior therapies, comorbidities, and center experience [2]. Furthermore, pivotal trials allowed only limited regimens, leaving a gap between trial constraints and the breadth of strategies used in practice [3–5]. The heterogeneity of approaches, the paucity of MM-specific prospective data, and evolving indications for earlier referral all argue for consensus-building and practice-informing evidence [6]. To address these uncertainties, we conducted a multinational survey and narrative review to characterize current practice patterns, rationales for regimen selection, and perceived risks/benefits of bridging prior to CAR-T for MM.

The survey was conceived to capture real-world practice on bridging therapy in MM patients undergoing CAR-T. Draft items were generated from consensus discussions among MM physicians, transplant/CAR-T program leads, and outcomes methodologists, mapped to prespecified domains (center characteristics; scope and timing of bridging; regimen selection drivers; monitoring and decision rules; pre-apheresis “holding” therapy; and open feedback). All supporting figures and data are shown in the Supplement. All methods were performed in accordance with the relevant guidelines and regulations by the EBMT.

In terms of characteristics of participants, across 48 responses from 11 countries, participation was led by Germany (33%) and Italy (21%). Most centers (58%) infused 10–30 MM patients per year. Product availability was uneven: cilta-cel was reported by 60%, ide-cel by 38%, while a notable 16% indicated no access outside clinical trials, underscoring an access bottleneck that persists across parts of Europe. In the German context, respondents emphasized a pragmatic constraint: only cilta-cel is reimbursed at present, a reality that shapes product mix and contributes to the broader European access challenge [7, 8].

In terms of bridging itself and the reality of its standardization and access, importantly, most programs report limited protocol standardization (from a scale of 1 “not standardized at all” to 5 “very standardized”): “not standardized at all” accounted for 40% of responses and only 4% selected 5 (“very standardized”). Most centers are not currently enrolled in trials or registries focused on bridging (65%).

Although bridging therapy was common: most centers reported bridging of >85% of their patients. Proteasome inhibitors were the most frequently reported bridging option (90%), followed by immunomodulatory drugs (IMiDs) (85%). Chemotherapy-based regimens and bispecific antibodies were each selected by 75.0%, while monoclonal antibodies were used by 69%, followed by radiation (63%). Importantly, of those centers who had experience with bispecific antibodies, all responded that bispecific antibodies should be standard for most patients, showing superior efficacy specifically for bridging.

In terms of duration, most centers described a typical bridging duration of 1–2 months (77%), with smaller shares reporting less than 1 month (15%). Initiation after apheresis is generally rapid: 81% begin immediately within one week. Together, these data depict a pattern of prompt initiation and a roughly one- to two-month treatment window for most programs.

First and foremost, most centers would still proceed to CAR-T after progression on bridging (irrespective of type of bridging). Overall, more than 95% continue toward infusion, with about four in ten opting to pause and reassess timing.

Regimen selection was driven primarily by prior therapy and refractory patterns (92%), followed by baseline disease burden (77%) and extramedullary disease (69%), with less emphasis on cytogenetic risk (31%), biochemical markers (21%), and bone marrow involvement (21%), underscoring a clear prioritization of treatment history and immediate disease burden over laboratory or marrow-based surrogates.

During bridging, monitoring relied predominantly on free light chains (96%) and serum M-protein (94%), with frequent use of imaging (PET/CT or MRI, 75%) and clinical symptom assessment (67%), less frequent bone marrow evaluation (35%), and rare cytogenetics (6%), while decision-making most often reflected achievement of targeted serum marker reductions (75%), signals of rapid disease progression (73%), treatment-related toxicities (54%), imaging improvements (52%), and, less commonly, reductions in marrow plasma cell burden (21%).

**Fig. 1:**
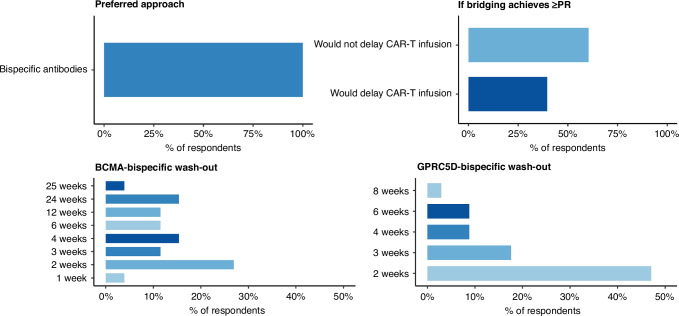
Bispecific bridging strategies.

In terms of special concerns regarding bispecific antibodies (Fig. 1), use of them for bridging showed short and variable wash-out practices, with BCMA-targeting agents most commonly held for 2 weeks (27%) but otherwise dispersed across 24 weeks (15%), 4 weeks (15%), 12 weeks (12%), 6 weeks (12%), 3 weeks (12%), 25 weeks (4%), and 1 week (4%), while GPRC5D-targeting agents were predominantly washed out for 2 weeks (47%) followed by 3 weeks (18%), 4 weeks (9%), 6 weeks (9%), and 8 weeks (3%), and when at least a partial hematologic response was achieved, most centers would not delay CAR-T infusion (60%).

Turning to holding therapy, most centers employed it to achieve rapid disease control in the setting of relapse or progression (60%) rather than primarily to debulk disease before collection (26%), while organizational delays were driven mainly by apheresis logistics (43%) rather than payer, trial, or industry constraints, two-thirds favored reusing the same regimen after apheresis (67%), bendamustine was almost universally considered contraindicated pre-collection (89%) with frequent caution toward bispecific antibodies (51%), and when bispecifics were used, programs generally recommended short two- to four-week wash-outs before apheresis despite substantial inter-center variability [9].

We then aimed to capture qualitative pictures of current concerns and consideration regarding bridging. In general, centers are trying to thread a narrow needle – achieving enough disease control to safely reach infusion while preserving T-cell fitness and not disqualifying patients through toxicity or timing missteps. The largest insecurity points are refractory biology, uneven access/reimbursement (especially for bispecifics), and logistics (manufacturing delays, step-up dosing, wash-outs). Respondents repeatedly asked for comparative, standardized evidence – clear guidance on regimen selection, radiotherapy’s role, optimal wash-outs (notably with talquetamab), and the effects of bridging on manufacturing, in-vivo expansion, and outcomes. Many favor avoiding pre-apheresis therapy when possible; when needed, bispecifics (often GPRC5D) or conventional chemotherapy are used, but concerns persist about BCMA target interference, lymphocyte exhaustion, and toxicity. Several advocate moving CAR-T earlier in the disease course and building registries to enable data-driven, less idiosyncratic bridging decisions.

In the absence of prospective comparative data on bridging strategies in MM, these findings point to pragmatic consensus practices, including broad avoidance of bendamustine before apheresis due to concerns about its lack of anti-MM efficacy, T-cell depletion and product fitness [10], selective but heterogeneous use of radiotherapy with calls for clearer indications and dosing standards, routine yet carefully tapered steroid use for rapid symptom control, and a preference for short, predictable cytoreductive chemotherapy regimens that minimize prolonged marrow suppression.

As bispecific antibodies are increasingly adopted for bridging, centers report two convergent practices: preferential use of non-BCMA targets when BCMA CAR-T is planned to mitigate concerns about antigen modulation or receptor occupancy, and generally short two- to four-week wash-outs despite heterogeneity, with growing recognition that bispecifics provide effective cytoreduction but carry risks of cytopenias and infection, and that once a partial hematologic response is achieved, most programs proceed directly to CAR-T rather than delay infusion in pursuit of deeper debulking.

In summary, this survey offers a real-world snapshot of bridging practices in relapsed/refractory multiple myeloma, showing that bridging is now an integral part of the CAR-T pathway, initiated rapidly after apheresis and typically sustained for one to two months, guided by highly individualized decision-making based on refractoriness, disease burden, and extramedullary involvement, and implemented using a broad and heterogeneous therapeutic armamentarium with limited protocol standardization and sparse trial or registry participation, underscoring a clear need for practice-informing evidence.

## Supplementary information

Supplement

## Data Availability

Survey data can be accessed from the corresponding author after reasonable request.

## References

[1] Fandrei D, Seiffert S, Rade M, Rieprecht S, Gagelmann N, Born P, et al. Bispecific antibodies as bridging to BCMA CAR-T cell therapy for relapsed/refractory multiple myeloma. Blood Cancer Discov. 2024. 10.1158/2643-3230.BCD-24-0118. 10.1158/2643-3230.BCD-24-0118 PMC11707513 39441177

[2] Richter J. Like a bridge over troubled water: keeping the myeloma down en route to CAR-T. Blood Cancer J. 2024;14:64. 10.1038/s41408-024-01049-z. 38609377 10.1038/s41408-024-01049-z PMC11015014

[3] Lin Y, Qiu L, Usmani S, Joo CW, Costa L, Derman B, et al. Consensus guidelines and recommendations for the management and response assessment of chimeric antigen receptor T-cell therapy in clinical practice for relapsed and refractory multiple myeloma: a report from the International Myeloma Working Group Immunotherapy Committee. Lancet Oncol. 2024. 10.1016/S1470-2045(24)00094-9. 38821074 10.1016/S1470-2045(24)00094-9

[4] Gagelmann N, Sureda A, Montoto S, Murray J, Bolanos N, Kenyon M, et al. Access to and affordability of CAR T-cell therapy in multiple myeloma: an EBMT position paper. Lancet Haematol. 2022;9:e786–e795. 10.1016/S2352-3026(22)00226-5. 36174641 10.1016/S2352-3026(22)00226-5

[5] Gagelmann N, Dima D, Merz M, Hashmi H, Ahmed N, Tovar N, et al. Development and validation of a prediction model of outcome after B-cell maturation antigen-directed chimeric antigen receptor T-cell therapy in relapsed/refractory multiple myeloma. J Clin Oncol. 2024:JCO2302232; 10.1200/JCO.23.02232. 10.1200/JCO.23.02232 PMC11095856 38358946

[6] Costa LJ, Banerjee R, Mian H, Weisel K, Bal S, Derman BA, et al. International myeloma working group immunotherapy committee recommendation on sequencing immunotherapy for treatment of multiple myeloma. Leukemia. 2025;39:543–54. 10.1038/s41375-024-02482-6. 39870767 10.1038/s41375-024-02482-6 PMC11879857

[7] Merz M, Albici AM, von Tresckow B, Rathje K, Fenk R, Holderried T, et al. Idecabtagene vicleucel or ciltacabtagene autoleucel for relapsed or refractory multiple myeloma: an international multicenter study. Hemasphere. 2025;9:e70070. 10.1002/hem3.70070. 39822585 10.1002/hem3.70070 PMC11735948

[8] Merz M, Gagelmann N, Smaili S, Flossdorf S, Sauer S, Scheid C, et al. Remission conversion drives outcomes after CAR T-cell therapy for multiple myeloma: a registry analysis from the DRST. Blood. 2025;146:1677–86. 10.1182/blood.2025028330. 40504993 10.1182/blood.2025028330

[9] Dhakal B, Akhtar OS, Fandrei D, Jensen A, Banerjee R, Pan D, et al. Sequential targeting in multiple myeloma: talquetamab, a GPRC5D bispecific antibody, as a bridge to BCMA CAR-T cell therapy. Blood. 2025. 10.1182/blood.2025029773. 40749169 10.1182/blood.2025029773

[10] Sidana S, Hosoya H, Jensen A, Liu L, Goyal A, Hovanky V, et al. Bendamustine vs. fludarabine/cyclophosphamide lymphodepletion prior to BCMA CAR-T cell therapy in multiple myeloma. Blood Cancer J. 2023;13:158. 10.1038/s41408-023-00929-0. 37833271 10.1038/s41408-023-00929-0 PMC10576036

