# Supplementary material for: Bridging therapy before CAR-T for multiple myeloma: a survey from the CMWP and CTIWP of the EBMT
Source: Bone Marrow Transplant. 2026 Mar 31;61(5):642–4. doi: 10.1038/s41409-026-02816-1 (PMC13152808; doi:10.1038/s41409-026-02816-1)

Supplement for:

**Bridging therapy before CAR-T for multiple myeloma: a survey from the CMWP and CTIWP of the EBMT**

Nico Gagelmann^1^*, Maximilian Merz^2,3^*, Laurien GA Baaij^4^, Linda Koster^4^, Jorinde D Hoogenboom^4^, Joanna Drozd-Sokolowska^5^, Kavita Raj^6^, Jürgen Kuball^7^, Patrick J Hayden^8^, Florent Malard^9^, Laurent Garderet^10^, Annalisa Ruggeri^11^, Donal McLornan^12^

1 University Medical Center Hamburg-Eppendorf, Hamburg, Germany

2 University Hospital Leipzig, Leipzig, Germany

3 Memorial Sloan Kettering Center, NYC, USA

4 EBMT Leiden Study Unit Leiden the Netherlands

5 University Clinical Centre, Medical University of Warsaw, Warsaw, Poland

6 University College London Hospitals NHS Trust, London, United Kingdom

7 University Medical Centre Utrecht, Utrecht, Netherlands

8 Department of Haematology, Trinity College Dublin, St. James’s Hospital, Dublin, Ireland

9 Hôpital Saint-Antoine, AP-HP, Paris, France

10 Hôpital Pitié Salpêtrière, Hematology department, Paris, France

11 IRCCS San Raffaele, Milano, Italy

12 University College London Hospitals NHS Trust, London, United Kingdom

*contributed equally

**Table 1. Synthesized open issues and challenges for bridging before CAR-T.**

| **Theme** | **Condensed issues** | **Illustrative notes from responses** |
| --- | --- | --- |
| Disease refractoriness and limited options | Many patients are triple/penta-refractory; picking an active regimen is difficult and often “trial-and-error.” | “multiple lines… reduced options and rapid progression”; “very few alternatives”; “patients refractory to almost all lines” |
| Balancing disease control vs toxicity & T-cell fitness | Need enough cytoreduction without exhausting lymphocytes or delaying CAR-T; concern that toxicity may render patients unfit. | “risk of causing toxicity… contraindicate CAR-T”; “right balance between toxicity sparing and effective tumor reduction” |
| Access, reimbursement & regulatory constraints | Access to drugs varies; reimbursement timing uncertain; some national rules restrict use (e.g., anti-BCMA BiAbs before anti-BCMA CAR-T). | “timely acknowledgment of reimbursement”; “not approved in 2nd line”; “availability (reimbursement)”; “in Italy… cannot be administered before anti-BCMA CAR-T” |
| Bispecific anibody concerns | Availability/reimbursement; timing (pre-apheresis vs bridging); target interference (BCMA), lymphocyte exhaustion; toxicity (e.g., weight loss/anosmia with GPRC5D); operational step-up/washouts. | “BCMA BiMab with BCMA CAR is not a good option”; “induced exhaustion… down-regulation of BCMA”; “GPRC5D weight loss, loss of smell” |
| Need for comparative/standardized evidence | Desire for guidelines and head-to-head/real-world data on which bridging works best, when to stop/switch, and impact on outcomes. | “CONSENSUS guidelines”; “data about best choice… and influence on CAR-T results”; “who profits from polychemotherapy like PACE?” |
| Center heterogeneity | Practices highly individualized by prior therapy, national rules, and resource constraints; some report few challenges. | “very individual”; “They are not frequent challenges” |
| Logistics and timing | Apheresis/manufacturing delays, inpatient capacity, wash-out/step-up dosing logistics. | “production time for CAR-T can be long”; “delay of hospitalization due to reduced capacities”; “wash-out period/step-up dosing logistics” |
| Specific evidence gaps | Radiotherapy role, optimal wash-outs (esp. talquetamab), biomarker guidance, impact on manufacturing/expansion and T-cell composition. | “More data on radiotherapy”; “wash-out of talquetamab”; “influence on product T-cell composition/expansion” |
| Preference signals | Several centers avoid pre-apheresis therapy if possible; when used, bispecifics (esp. GPRC5D) or conventional chemo can be effective with careful wash-outs. | “we do not administer BiAbs as bridging before apheresis”; “good experience with talquetamab or chemo”; “pre-apheresis should be avoided” |
| Earlier-line CAR-T | Belief that moving CAR-T earlier would reduce refractoriness and simplify bridging. | “CAR-T in earlier lines… less refractory patients” |
| Data infrastructure & registries | Interest in participating in studies/registries; calls for data-driven selection tools. | “We are very interested in participating”; “data driven treatment selection” |

Figure 1


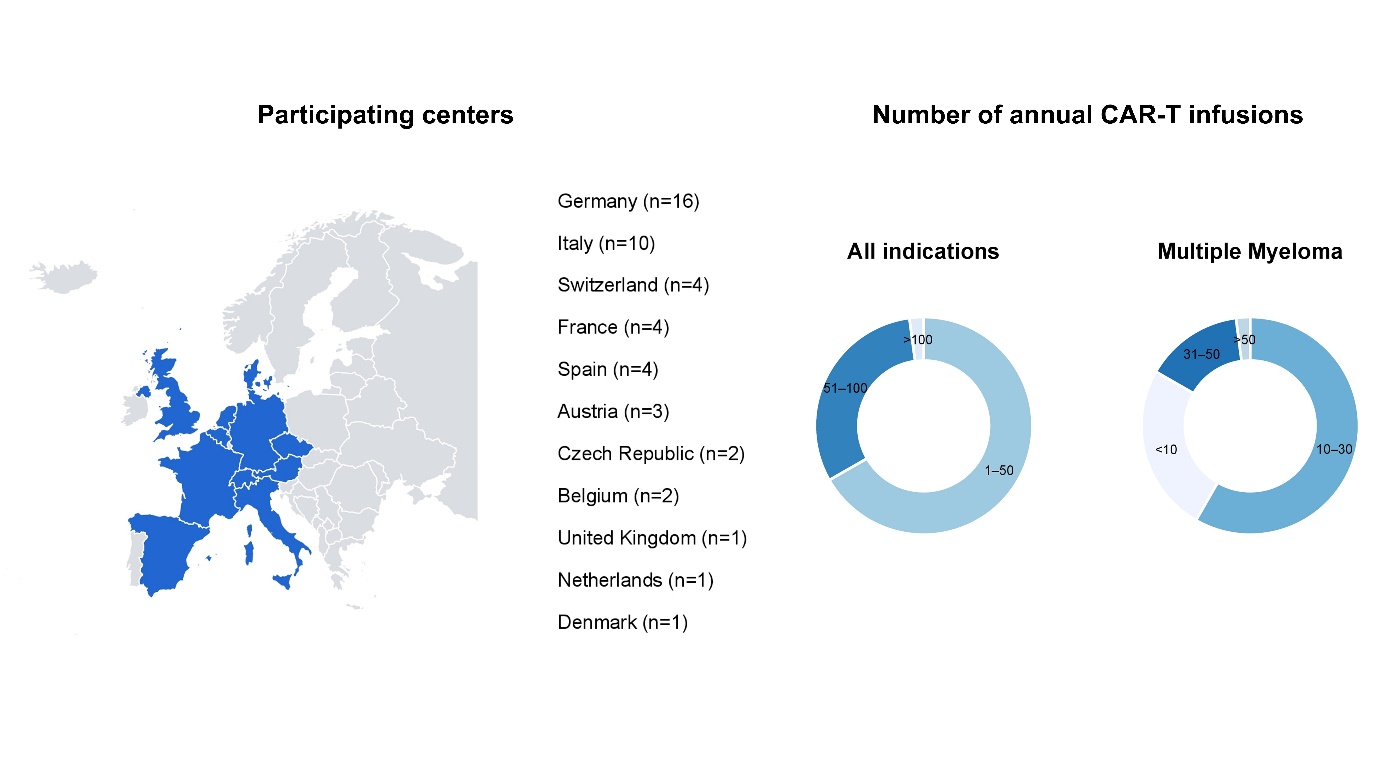


Figure 2


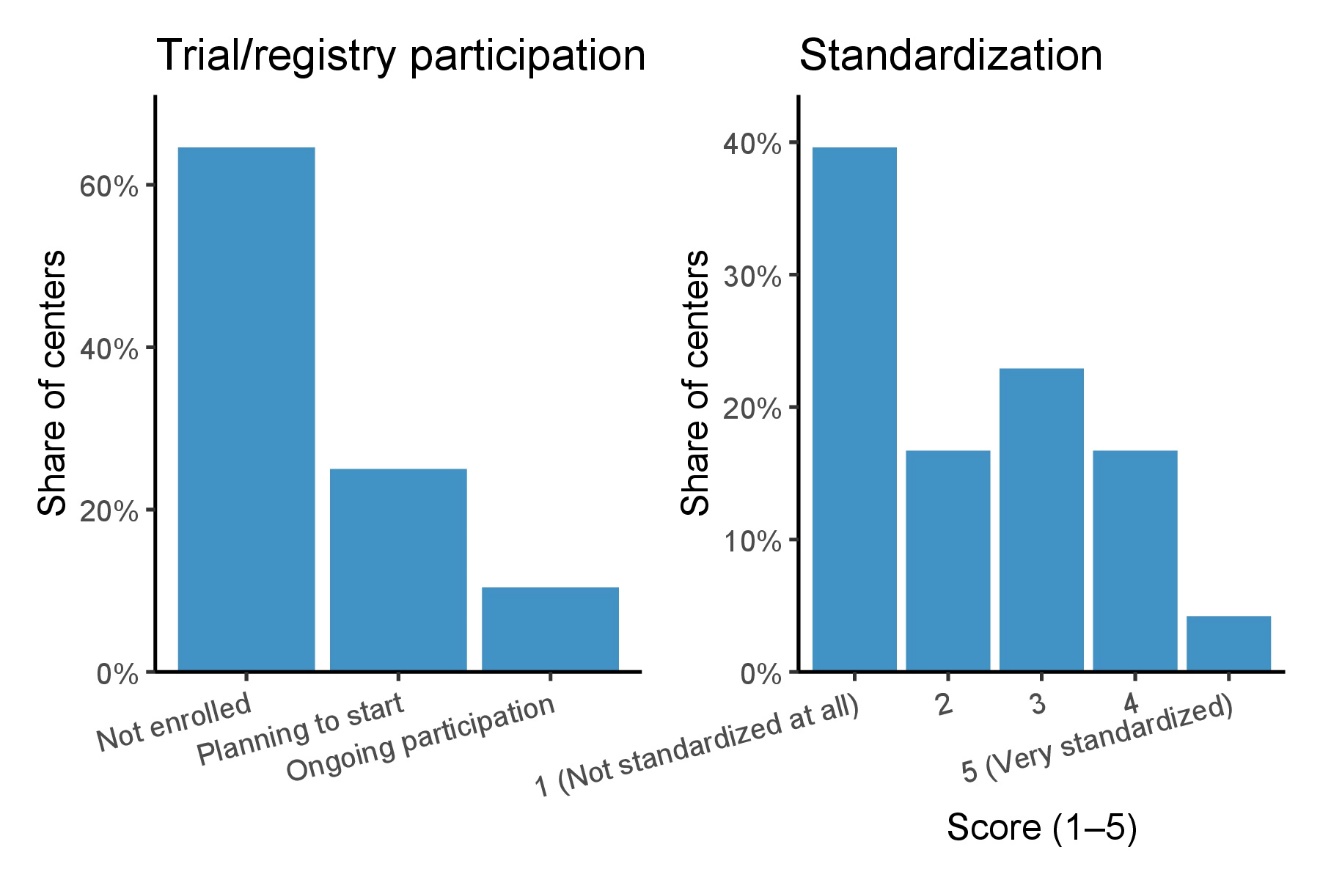


Figure 3


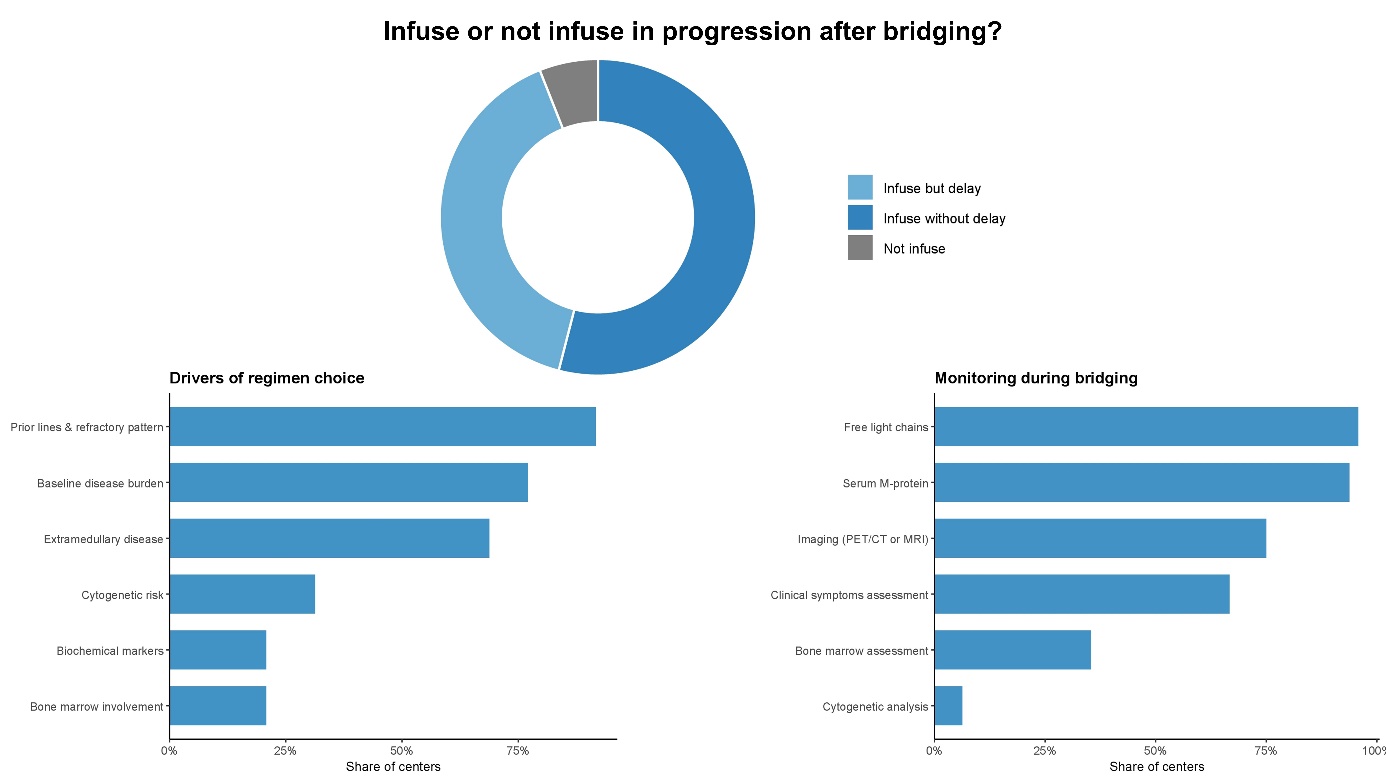

Supplement: Supplementary file 1 — Supplement [file 41409_2026_2816_MOESM1_ESM.docx]
